# Diabetes mellitus – risk factor and potential future target for hepatic encephalopathy in patients with liver cirrhosis?

**DOI:** 10.1007/s11011-022-01068-4

**Published:** 2022-08-24

**Authors:** Simon Johannes Gairing, Eva Maria Schleicher, Christian Labenz

**Affiliations:** 1 Department of Internal Medicine I, University Medical Center of the Johannes Gutenberg-University, Langenbeckstrasse 1, 55131 Mainz, Germany; 2 Cirrhosis Center Mainz (CCM), University Medical Center of the Johannes Gutenberg- University, Mainz, Germany

**Keywords:** Insulin, Glucose, Chronic liver disease, HE, Type 2 diabetes mellitus

## Abstract

Hepatic encephalopathy (HE) is one of the major complications of cirrhosis, and its presence is associated with poor survival. Several risk factors for HE are well established, including age, history of HE, portosystemic shunts, or poorer liver function. In recent years, diabetes mellitus (DM) has emerged as another potential risk factor for the development of HE. This may be important for many patients, as the incidence of type 2 DM (T2DM) is increasing worldwide and, consequently, the incidence of NAFLD-related cirrhosis is rising simultaneously. In addition, DM is a critical factor in the progression of other liver diseases, such as alcohol-related liver disease. Thus, the number of patients with cirrhosis and comorbid T2DM will also increase. To date, the prevalence of DM already ranges between 22 - 40% in patients with cirrhosis. DM-associated factors that may influence the risk of HE include systemic inflammation, insulin resistance with increased muscle protein breakdown as well as autonomic dysfunction with prolonged intestinal transit time and small intestinal bacterial overgrowth. Currently, the evidence for an association between DM and both minimal and overt HE is weak and it seems likely that only poor glycemic control has an impact on HE risk. In addition, there are some early signs indicating that DM may impair the response of patients with HE to pharmacological therapies such as rifaximin. Thus, improvements in the management of glycemic control may be a candidate future target to reduce the risk of HE. In this concise review, we summarize the current evidence on the association between DM and HE and its potential future implications.

## Introduction

Hepatic encephalopathy (HE) is a critical complication in patients with acute liver failure (ALF), acute-on-chronic liver failure (ACLF), or decompensated cirrhosis, and is associated with poor prognosis (Cordoba et al. 2014; Rose et al. 2020). Roughly, HE can be described as a complex neurocognitive disorder caused by an impairment of the hepatic ability to detoxify neurotoxic metabolites and/or portosystemic shunting (Vilstrup et al. 2014).

**Fig. 1:**
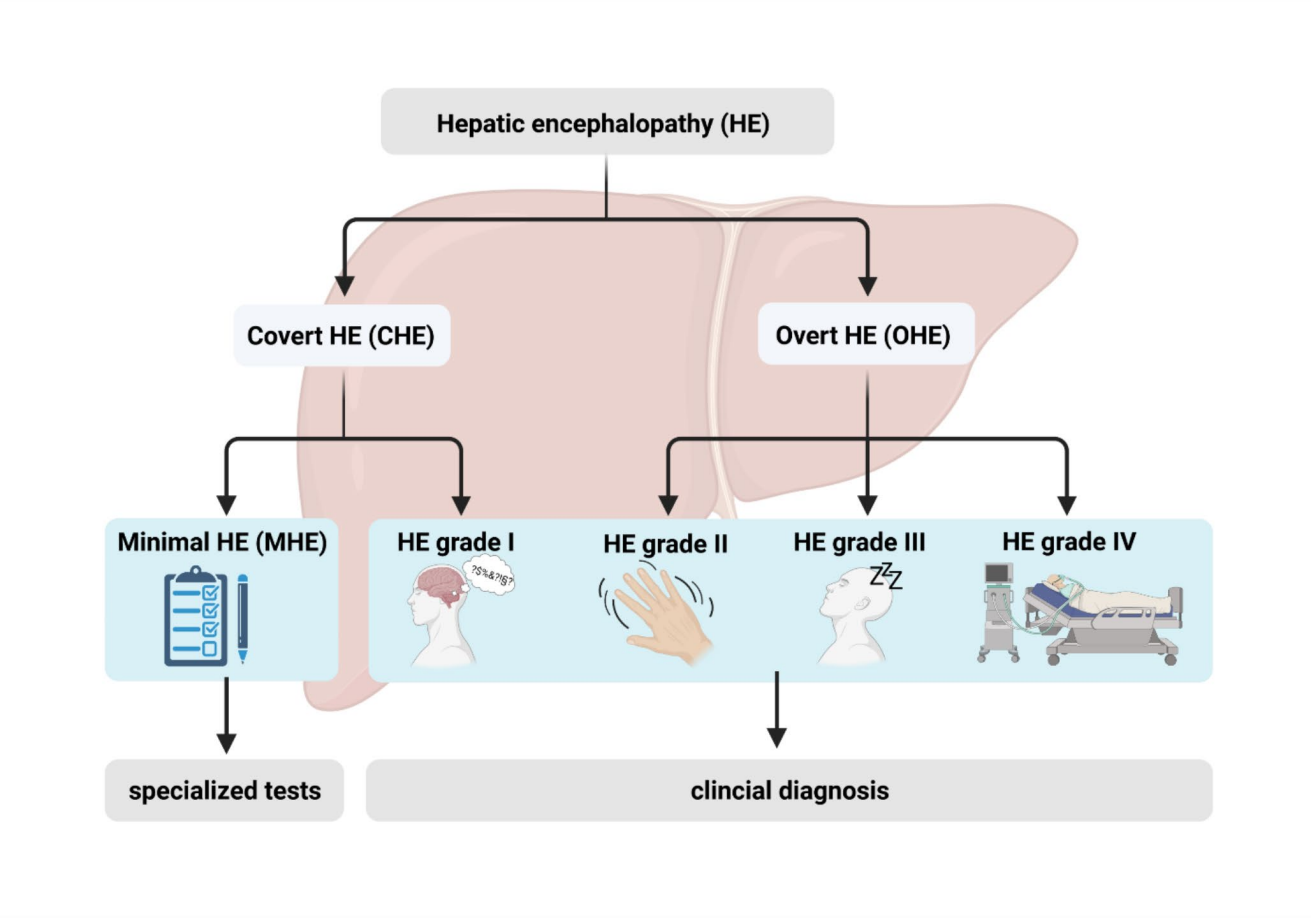
**Grading of hepatic encephalopathy (HE) according to the West-Haven criteria.** Minimal hepatic encephalopathy (MHE) is the lowest HE grade and can only be diagnosed by psychometric testing. HE grades 1–4 are clinically detectable. CHE: covert hepatic encephalopathy; OHE: overt hepatic encephalopathy. Created with BioRender.com

Symptoms of HE range from low-grade cognitive impairment to coma. HE is subdivided into two main categories: covert HE (CHE) and overt HE (OHE) (Vilstrup et al. 2014). CHE comprises the two lowest grades according to the West-Haven criteria (minimal hepatic encephalopathy (MHE) and HE grade 1 (HE 1)), whereas OHE includes the HE grades 2 to 4 (HE 2–4). Figure 1 displays the classification of HE according to the West-Haven criteria.

Major HE risk factors include age, poorer liver function, a history of OHE or insertion of a transjugular intrahepatic portosystemic shunt (TIPS) (Gairing et al. 2022b; Rose et al. 2020). A growing body of evidence indicates that several underlying etiologies that drive chronic liver disease have direct effects on the brain and may be important contributors to the individual HE risk in the long term (Balzano et al. 2018; Forton et al. 2001; Grover et al. 2016; Mosher et al. 2018; Rose et al. 2020). In addition, diabetes mellitus (DM) has been identified as another potential risk factor for the development of HE in patients with cirrhosis (Elkrief et al. 2014; Jepsen et al. 2015; Kalaitzakis et al. 2007; Labenz et al. 2020a). In general, DM is associated with a higher risk of cirrhosis-associated complications and poorer survival in patients with cirrhosis (Elkrief et al. 2014, 2016). In recent decades, the prevalence of type 2 diabetes mellitus (T2DM) has increased worldwide and represents an unprecedented public health burden (Tinajero and Malik 2021). DM itself has detrimental effects even on patients without liver cirrhosis and is one of the most important risk factors for developing non-alcoholic fatty liver disease (NAFLD) (Friedman et al. 2018). This is one of the reasons why the incidences of NAFLD, NAFLD-related cirrhosis and ultimately NAFLD-related hepatocellular carcinoma (HCC) are rising (Foerster et al. 2022). Additionally, DM plays an important role not only in the development of NAFLD-associated cirrhosis, but also in patients with cirrhosis of other etiologies, such as alcohol-related cirrhosis (Whitfield et al. 2022; Wlazlo et al. 2010). Thus, the number of patients with both cirrhosis and DM will likely increase. To date, the prevalence of DM already ranges from 22 to 40% in patients with cirrhosis (Butt et al. 2013; Elkrief et al. 2014; Jepsen et al. 2015). In light of this development, knowledge on the influence of DM on HE is critical, as glycemic control is a potentially modifiable target to prevent HE. In recent years, several articles reviewed the impact of DM and/or NAFLD on cognitive function (Biessels and Despa 2018; Colognesi et al. 2020; Kjærgaard et al. 2021; Weinstein et al. 2018). In this article, we expand these articles by reviewing the current knowledge on the association between DM and the risk of HE in patients with cirrhosis.

## Pathophysiology of HE in patients with cirrhosis and diabetes mellitus

The pathophysiology of HE is complex and still not fully understood. Roughly, it is based on a combination of hyperammonemia and systemic inflammation. The detailed pathophysiology of HE in patients with cirrhosis is comprehensively reviewed elsewhere and not within the scope of this article (Rose et al. 2020).

**Fig. 2:**
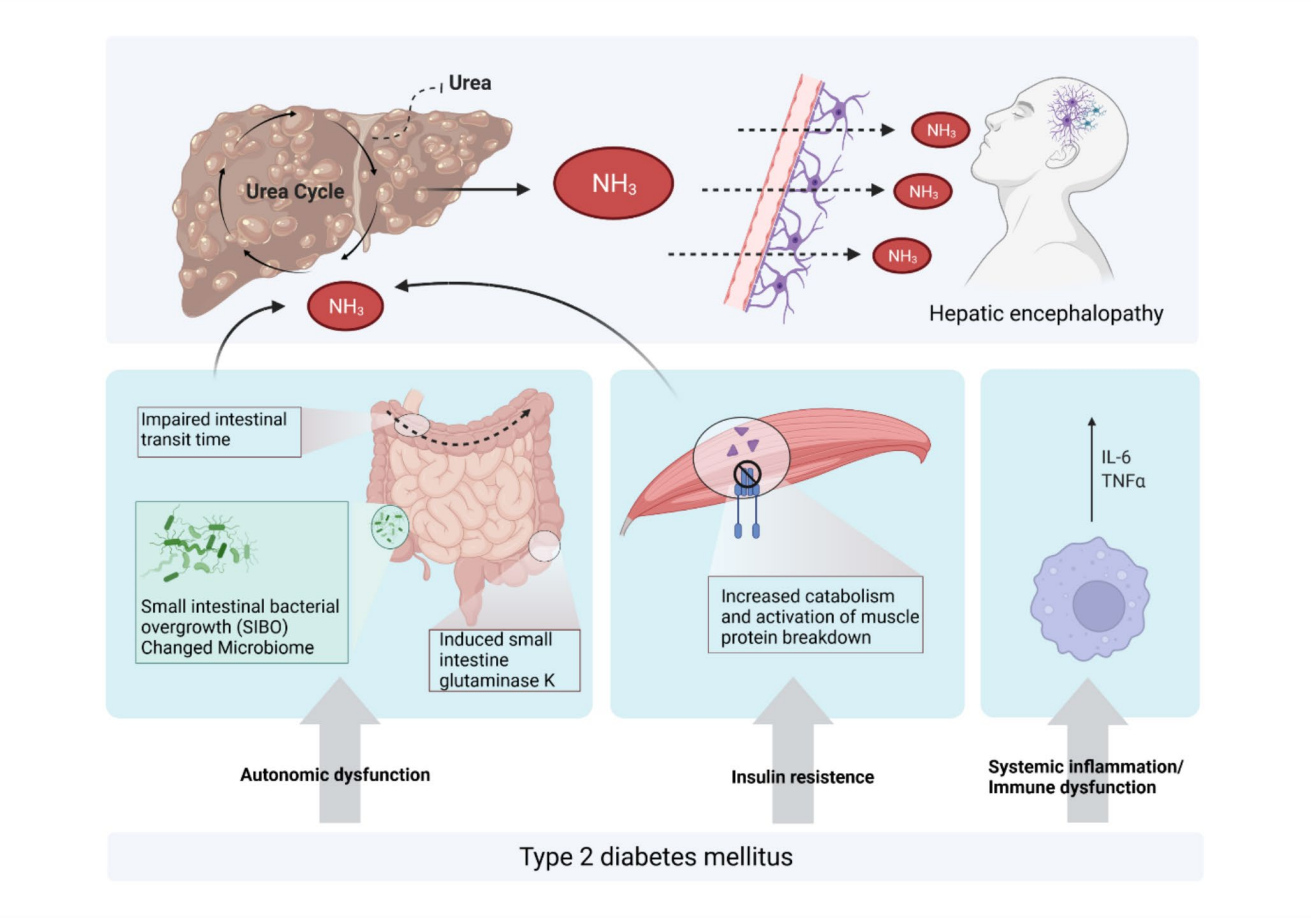
**Diabetes-associated factors which may trigger or exacerbate hepatic encephalopathy (HE).** DM-related factors that may influence the risk of HE include autonomic dysfunction with prolonged intestinal transit time and small intestinal bacterial overgrowth (SIBO), insulin resistance with increased muscle protein breakdown, and systemic inflammation due to increased production of pro-inflammatory cytokines such as interleukin-6 (IL-6) or tumor-necrosis factor alpha (TNFα). NH3: ammonium hydroxide. Created with BioRender.com

There are several effects of DM that may link DM and HE in patients with cirrhosis (Fig. 2). First, DM is associated with autonomic dysfunction. As a result, the gastrointestinal transit time is delayed, which promotes constipation and small intestinal bacterial overgrowth (SIBO) (Abrahamsson 1995). Even hyperglycemia itself impairs gastric and small intestinal motility leading to a prolonged gastrointestinal transit time. This may elevate the risk of HE in patients with cirrhosis by an increase in bacterial translocation from the gut and an increased intestinal ammonia production (Sigal et al. 2006; Spengler et al. 1989; Wegener et al. 1990). Second, DM is associated with alterations in the gut microbiome itself (Qin et al. 2012). Third, DM could increase ammonia production by inducing small intestine glutaminase type K and accelerating muscle breakdown (Ampuero et al. 2012). Fourth, T2DM-associated insulin resistance (IR) may promote an increased protein catabolism, which may result in higher blood ammonia levels. In this context, Chow et al. found that insulin does not stimulate muscle protein synthesis, but causes muscle protein anabolism by inhibiting muscle protein breakdown (Chow et al. 2006). Insulin also acts on the central nervous system (CNS) by modulating behavior and systemic metabolism (Kullmann et al. 2020). Here, it promotes neurite growth, modulates catecholamine release and uptake, regulates ligand-gated ion channel transport, GABA expression and localization, N-methyl-d-aspartate (NMDA) and α-amino-3-hydroxy-5methyl-4-isoxazolepropionic acid (AMPA) receptors. This results in a modulation of activity-dependent synaptic plasticity, i.e., long-term potentiation (LTP) and long-term depression (LTD) via NMDA receptor signaling (Arnold et al. 2018; Fadel and Reagan 2016; van der Heide et al. 2005). Thus, IR of certain brain areas, including the hypothalamus, the hippocampus and the cerebral cortex, has a negative effect on metabolism by disturbing cerebral insulin pathways. Moreover, it is well known that the consumption of a high caloric diet also leads to high concentration of inflammatory cytokines in the brain, resulting in microgliosis, astrocytosis and neuronal damage (Bélanger et al. 2011; Horvath et al. 2010). These pathophysiological aspects might play a role not only in HE-related but also in HE-independent cognitive deficits caused by DM and glycemic control.

Fifth, systemic inflammation not only plays a critical role in T2DM, but also in the development of HE (Tranah et al. 2013). In this context, IR and T2DM are considered as chronic inflammatory conditions due to the increased production of pro-inflammatory cytokines such as interleukin-6 (IL-6) or tumor-necrosis factor alpha (TNFα) (Basu et al. 2011). Recently, increased IL-6 serum levels were found to be associated with the presence of MHE and the development of OHE (Gairing et al. 2022a; Labenz et al. 2019). Last, both T2DM and cirrhosis increase the susceptibility of patients to bacterial infections, which may further enhance the release of pro-inflammatory cytokines (Diaz et al. 2008; Trail et al. 1993).

## Cognitive impairments in patients with diabetes mellitus without cirrhosis

The link between DM, cognitive deficits, and the development of dementia is well established in the literature (You et al. 2021; Zilliox et al. 2016). In this context, passive cognitive impairment triggered by acute hypo- or hyperglycemia, persistent mild cognitive impairment, and clinically relevant dementia should be differentiated. In general, elevated blood glucose levels are associated with a deterioration in cognitive performance in the long term (Tortelli et al. 2017). This is underscored by a study by Morris et al. suggesting that glycemic control is related to cognitive decline and progression to Alzheimer’s Disease (AD) (Morris et al. 2014). Moreover, T2DM is associated with a 50% increase in the risk of dementia (Biessels et al. 2006). To date, it is unclear whether factors related to glycemic dysregulation increase dementia risk or whether this risk is modulated by other DM-associated factors, particularly an unfavorable vascular risk factor profile (Sharma et al. 2020). Ultimately, several factors are involved that individually may only have a small effect on their own, but collectively can cause cognitive dysfunction (Biessels and Despa 2018). Prominent examples are atherosclerosis and stroke, as well as ischemic small vessel disease, which are more common in patients with T2DM than in the general population (Abner et al. 2016). In addition, patients with manifestations of microvascular (e.g., diabetic retinopathy) or macrovascular disease (e.g., myocardial infarction, stroke) are also more likely to have poorer cognitive performance compared to healthy individuals (Biessels et al. 2014; Feinkohl et al. 2015).

The pathophysiology of diabetic cognitive impairment is complex but is likely to involve the following mechanisms: abnormalities in insulin signaling and insulin receptor sensitivity in neurons, dysregulation and defects of mitochondria in the presynaptic hippocampus, diabetic autonomic neuropathy and an increase in the expression of pro-inflammatory cytokines in the brain (Zilliox et al. 2016). In addition, microcirculatory disorders, such as disruption of the blood-brain barrier (BBB), also play an important role in the development of cognitive impairment (Mogi and Horiuchi 2011). This change in permeability could lead to lower insulin levels in the brain and lower insulin-supported neuronal and glial activity (Heni et al. 2014).

In T2DM, cognitive changes mainly affect learning and memory, mental flexibility and speed (Awad et al. 2004; Stewart and Liolitsa 1999; Tortelli et al. 2017). In general, early diagnosis of cognitive impairment is critical to ensure that patients are still able to independently and reliably manage (insulin-) therapy and the appropriate diet. Thus, patients with T2DM and signs of cognitive deficits should be screened annually for dementia using established tests (Kulzer et al. 2013).

## Diabetes mellitus and minimal hepatic encephalopathy (MHE)

MHE is the lowest grade of HE and can only be detected with specialized tests. The portosystemic hepatic encephalopathy score (PHES) is the current gold standard for MHE diagnosis (Vilstrup et al. 2014). Patients with HE 1 only show low-grade clinical symptoms. Thus, diagnosis of HE 1 is examiner-dependent and often not reliably reproducible (Reuter et al. 2018). About a decade ago, MHE and HE 1 were grouped together under the umbrella term “covert” HE (CHE). CHE affects approximately one-third of all patients with cirrhosis and has a distinct impact on patients’ quality of life as well as their prognosis (Labenz et al. 2018, 2020b). Currently, only few studies investigated the association between DM and the prevalence of MHE or CHE (Table 1). Kalaitzakis et al. found an association between the time to conduct the number connection test A (NCT A), which is a part of PHES, and the presence of DM (Kalaitzakis et al. 2007). When interpreting this study, it has to be acknowledged that the NCT A is no validated tool to detect MHE in patients with cirrhosis. Nevertheless, a recently published study found a moderate association between DM and the presence of CHE, defined by the gold standard PHES, in a large cohort of patients with cirrhosis (Labenz et al. 2020a). However, it has to be mentioned that this association lost significance when excluding patients with a history of OHE from the multivariable analysis. One of the most important findings of this study was that there was a significant association between HbA1c levels ≥ 6.5% and the presence of CHE, while there was no significant association between HbA1c levels < 6.5% and CHE (Labenz et al. 2020a). This finding underscores the hypothesis that there may not be a relationship between DM and CHE per se, but that glycemic control may be the relevant modulator.

**Table 1:** Studies evaluating the association between diabetes mellitus and covert hepatic encephalopathy (CHE)

Study	Study design	Study population	n (%) with DM	Results
(Acharya et al. 2021)	Prospective	n = 700 outpatients with cirrhosis	237 (33.9%)	No association between DM and CHE
(Labenz et al. 2020a)	Prospective	n = 240 with cirrhosis	65 (27.1%)	DM independently associated with CHE. Especially patients with HbA1c levels ≥ 6.5% were at higher risk for CHE (OR 2.264, 95% CI 1.002–5.118)
(Kalaitzakis et al. 2007)	Prospective	n = 128 with cirrhosis	33 (26%)	DM independently associated with an increased time needed for number connection test A

Abbreviations: DM, diabetes mellitus; CHE, covert hepatic encephalopathy; HbA1c, hemoglobin A1c

There are also negative studies that argue against an association between DM and MHE/CHE. A recent study by Acharya et al. investigating one of the largest cohorts ever published in the context of CHE (700 patients) found no association between major comorbidities, such as DM, and the presence of CHE (Acharya et al. 2021).

It has to be acknowledged that all of the aforementioned studies are limited by their cross-sectional design. Therefore, prospective studies with serial testing are needed in the future to determine whether patients with DM are at higher risk for the development of MHE/CHE. Another fact that has to be kept in mind when interpreting the aforementioned studies is that the tests used for detection of MHE are not specific for HE. Therefore, the interpretation of the respective test results may be complicated by the coexistence of other diseases leading to chronic brain injury, such as dementia or chronic alcohol consumption. As already reviewed above, DM may alter cognitive function and especially affects domains such as attention, memory, executive function, processing speed or motor dysfunction (Weissenborn 2019). Some of these changes overlap with dysfunctions caused by HE and may therefore hamper the interpretation of MHE tests. A study by Lauridsen et al. investigated the impact of common chronic diseases, including DM, on results in the PHES as well as the continuous reaction time (CRT) (Lauridsen et al. 2016). In this study, they found that the results in PHES or CRT did not differ between patients with DM or healthy controls. However, it has to be mentioned that 3 of 15 patients with DM had results of -4 and below in the PHES. Another study conducted by Goldbecker et al. found no influence of DM neither on PHES, nor on inhibitory control test (ICT) (Goldbecker et al. 2013). In summary, the current evidence does not suggest that DM may significantly interfere with MHE testing. However, additional studies in larger populations are needed to further elucidate this important topic. In particular, there is an urgent need for studies investigating the impact of DM on screening tests for MHE such as the simplified animal naming test (S-ANT1), which may be more broadly used in routine clinical practice (Campagna et al. 2017). Due to the fact that the S-ANT1 also tests memory, a domain that may not be directly affected by HE, the influence of diabetes mellitus on test results may be even stronger than that of HE (Weissenborn et al. 2003).

## Diabetes mellitus and overt hepatic encephalopathy (OHE)

The development of OHE is a turning point in the course of cirrhosis and a remarkable predictor of poor prognosis (Jepsen et al. 2010). In addition to poor prognosis, OHE has detrimental effects on patients’ quality of life, is a huge burden for caregivers and is associated with a cumulative cognitive deficit (Bajaj et al. 2010; Nagel et al. 2022). In contrast to several liver-related risk factors, the impact of the most common comorbidities of patients with cirrhosis, such as DM, on the risk of OHE has not been extensively studied in the past (Labenz et al. 2020b; Praktiknjo et al. 2020). Additionally, most of the published studies are of comparably low evidence and either retrospective or cross-sectional in design. A study conducted by Sigal et al. investigated the impact of DM on the presence of different HE grades in a relatively small subset of patients with hepatitis C virus-associated cirrhosis (Sigal et al. 2006). Although the association between DM and the presence of HE did not reach significance, patients with DM suffered from more severe HE grades. Currently, there are only two prospective studies describing an association between DM and the development of OHE. The first one is a post-hoc analysis of the satavaptan trials in patients with cirrhosis and ascites. In this study, Jepsen et al. found a robust association between DM and the development of OHE with an adjusted hazard ratio of 1.86 (Jepsen et al. 2015). However, this study is limited by the lack of CHE testing at baseline. The second study included 240 prospectively followed patients of whom 27% suffered from DM (Labenz et al. 2020a). This study found an association between DM and the development of OHE even after adjusting for the presence of CHE at baseline in multivariable analysis. Again, as also mentioned above, this study demonstrated that especially the degree of glycemic control seems to be associated with the development of OHE rather than DM per se. Here, the OHE-rate was increased in patients with poorer glycemic control (HbA1c ≥ 6.5% vs. no diabetes mellitus), while there was no association between better glycemic control (HbA1c < 6.5%) and the development of OHE (Labenz et al. 2020a). However, it has to be mentioned that the findings of this study are limited by its observational design and the single-center setting. To give a balanced view on the impact of DM on the risk for OHE, it has to be reported that there are also negative studies reporting on this topic. A recently published study by Acharya et al. investigated the association between comorbid conditions and the development of OHE in a large cohort of patients with cirrhosis (700 patients, 33% with prior OHE) (Acharya et al. 2021). Here, only liver-related variables predicted the occurrence of OHE in patients with prior OHE, while DM turned out to be no risk factor. Table 2 gives a brief overview on relevant studies investigating a potential association between DM and OHE.

**Table 2:** Studies investigating the association between diabetes mellitus and overt hepatic encephalopathy (OHE)

Study	Study design	Study population	n (%) with DM	Results
(Acharya et al. 2021)	Prospective	n = 700 outpatients with cirrhosis	237 (33.9%)	No association between DM and time to OHE-related readmission
(Labenz et al. 2020a)	Prospective	n = 240 with cirrhosis	65 (27.1%)	DM independently associated with development of OHE. Especially patients with HbA1c levels ≥ 6.5% were at higher risk for OHE (HR 4.116, 95% CI 1.791–9.459)
(Jepsen et al. 2015)	Post-hoc analysis from 3 RCTs	n = 862 with cirrhosis and ascites	193 (22%)	aHR 1.86 (95% CI 1.20–2.87) for new-onset OHE (diabetic + vs. diabetic-)
(Elkrief et al. 2014)	Retrospective	n = 348 patients with HCV-associated cirrhosis	139 (40%)	DM independently associated with presence of HE
(Butt et al. 2013)	Prospective	n = 352 with decompensated cirrhosis	118 (33.5%)	Baseline DM independently associated with HE at baseline (OR 6.551, 95% CI 2.600-16.504)
(Gundling et al. 2013)	Retrospective	n = 285 with cirrhosis	87 (30.5%)	DM independently associated with HE occurence (OR_adj_ 3.21,95% CI: 1.63–6.28)
(Sigal et al. 2006)	Cross-sectional	n = 65 with HCV-associated cirrhosis	20 (31%)	DM associated with more severe HE stages

Abbreviations: DM, diabetes mellitus; OHE, overt hepatic encephalopathy; RCT, randomised controlled trial; HE, hepatic encephalopathy; aHR, adjusted hazard ratio; HCV, hepatitis-C virus; ORadj, adjusted odds ratio

In conclusion, DM could be a risk factor for the development of OHE. However, the current evidence is only mediocre and based on cross-sectional studies, cohort studies and a post-hoc analysis of randomized-controlled trials with a focus on ascites. Therefore, additional high-quality studies are needed to draw a definitive conclusion on this important topic.

## Therapeutic approaches

Considering the increasing prevalence of patients with cirrhosis with comorbid DM and its potential association with HE, DM might be a future therapeutic target for reducing the risk of HE. As mentioned above, poorer glycemic control seems to be associated with a higher OHE-risk (Labenz et al. 2020a). However, there is currently no prospective study available investigating the effect of an improvement in glycemic control on HE-risk. A study by Ampuero et al. demonstrated in a preclinical model that metformin inhibits glutaminase activity and may therefore have the potential to prevent HE (Ampuero et al. 2012). This hypothesis was validated in a retrospective analysis of 82 patients with cirrhosis. Here, metformin intake was associated with a lower risk of OHE during follow-up. However, these findings are in contrast to a recently published study indicating that metformin is associated with a reduced responsiveness to rifaximin (Ballester et al. 2022). In the future, prospective and randomized trials are needed to investigate these associations in more detail.

In an older study, acarbose was found to significantly reduce ammonia serum levels and to improve cognitive function in patients with cirrhosis, T2DM and HE grade 1–2 in a randomized controlled trial (Gentile et al. 2005). However, there are some case reports on fulminant hepatitis in patients treated with acarbose (Diaz-Gutierrez et al. 1998; La Vega et al. 2000). In addition, acarbose may lead to hypoglycemia, which may have worrisome consequences in patients with decompensated cirrhosis. Thus, acarbose is currently contraindicated in patients with cirrhosis.

In addition to studies indicating DM as a potential target for reducing the risk of HE, there is also preliminary evidence suggesting potentially harmful effects of DM on established HE treatments. Recently, a bicentric prospective cohort study including 63 patients with cirrhosis investigated the association of the metabolic syndrome on the response to rifaximin in patients with MHE (Ballester et al. 2022). The study found that older age, NAFLD, all metabolic syndrome-associated diseases including DM and metformin intake were associated with a reduced responsiveness to rifaximin (Ballester et al. 2022). However, the results of this study have to be interpreted in the context of its design. The sample size is comparably small and patients were not randomized to treatment. Additionally, the adherence rate to rifaximin treatment was only 72% and not surprisingly adherence to therapy was significantly associated with a better response to therapy. Therefore, more studies on this topic are needed before definitive conclusions can be drawn regarding the impact of DM on treatment with rifaximin.

In general, treatment of DM in patients with cirrhosis is challenging. First, lifestyle modifications such as diets (e.g., in obese patients with NAFLD-related cirrhosis) have to be finely balanced to achieve weight loss without triggering HE due to catabolism. Sarcopenia is a well-established risk factor for HE and should not be aggravated due to diet restrictions (Nardelli et al. 2019). Second, usually it is not easy to implement sport interventions into the daily routine of patients with decompensated cirrhosis (Elkrief et al. 2016). Last, most medications used for the treatment of DM are not approved in patients with impaired liver function. Metformin appears to be safe in more or less compensated patients with cirrhosis but it is contraindicated in concurrent acute kidney injury such as hepatorenal syndrome (HRS-AKI) (Elkrief et al. 2016). Regarding other pharmacological agents, their individual hepatotoxic potency and risk of hypoglycemia have to be kept in mind.

## Conclusion and future perspectives

DM is a frequent comorbidity in patients with cirrhosis. Several studies indicate an association between diabetes mellitus as well as poorer glycemic control and the development of HE, although there are also conflicting results. However, studies are frequently limited by their designs, small cohorts and varying diagnostic tools and/or definitions used for diagnosing HE. Therefore, larger multicenter studies with sufficient follow-up and longitudinal assessment of cognitive function and glycemic control are urgently needed to clear the dust on this important topic.

In conclusion, the reciprocal relationship between cognitive impairment, long-term inadequate adherence to therapy, and unsatisfactory metabolic control may have the effect of a vicious circle. Therefore, it is of pivotal importance to screen patients with cirrhosis for new-onset DM and to adequately treat patients with an established diagnosis.

## Data Availability

No new data were generated for this review.

## Abbreviations

ALF: Acute liver failure
ACLF: acute-on-chronic liver failure
CHE: covert HE
DM: diabetes mellitus
HE: hepatic encephalopathy
HCC: Hepatocellular Carcinoma
IR: insulin resistence
IL-6: interleukin-6
MHE: minimal hepatic encephalopathy
NCT A: number connection test A
NAFLD: non-alcoholic fatty liver disease
OHE: overt HE
PHES: portosystemic hepatic encephalopathy score
S-ANT1: simplified animal naming test
T2DM: type 2 diabetes mellitus
TNF: tumor-necrosis factor alpha
TIPS: transjugular intrahepatic portosystemic shunt

## References

[CR1] Abner EL, Nelson PT, Kryscio RJ, Schmitt FA, Fardo DW, Woltjer RL, Cairns NJ, Yu L, Dodge HH, Xiong C, Masaki K, Tyas SL, Bennett DA, Schneider JA, Arvanitakis Z (2016). Diabetes is associated with cerebrovascular but not Alzheimer’s disease neuropathology. Alzheimers Dement.

[CR2] Abrahamsson H (1995). Gastrointestinal motility disorders in patients with diabetes mellitus. J Intern Med.

[CR3] Acharya C, Nadhem O, Shaw J, Hassouneh R, Fagan A, McGeorge S, Sterling RK, Puri P, Fuchs M, Luketic V, Sanyal AJ, Wade JB, Gilles HS, Heuman DM, Tinsley F, Matherly S, Lee H, Siddiqui MS, Thacker LR, Bajaj JS (2021). Liver-Unrelated Comorbid Conditions Do Not Affect Cognitive Performance or Hepatic Encephalopathy Progression in Cirrhosis. Am J Gastroenterol.

[CR4] Ampuero J, Ranchal I, Nuñez D, Del Díaz-Herrero MM, Maraver M, del Campo JA, Rojas Á, Camacho I, Figueruela B, Bautista JD, Romero-Gómez M (2012). Metformin inhibits glutaminase activity and protects against hepatic encephalopathy. PLoS ONE.

[CR5] Arnold SE, Arvanitakis Z, Macauley-Rambach SL, Koenig AM, Wang H-Y, Ahima RS, Craft S, Gandy S, Buettner C, Stoeckel LE, Holtzman DM, Nathan DM (2018). Brain insulin resistance in type 2 diabetes and Alzheimer disease: concepts and conundrums. Nat Rev Neurol.

[CR6] Awad N, Gagnon M, Messier C (2004). The relationship between impaired glucose tolerance, type 2 diabetes, and cognitive function. J Clin Exp Neuropsychol.

[CR7] Bajaj JS, Schubert CM, Heuman DM, Wade JB, Gibson DP, Topaz A, Saeian K, Hafeezullah M, Bell DE, Sterling RK, Stravitz RT, Luketic V, White MB, Sanyal AJ (2010). Persistence of cognitive impairment after resolution of overt hepatic encephalopathy. Gastroenterology.

[CR8] Ballester M-P, Gallego J-J, Fiorillo A, Casanova-Ferrer F, Giménez-Garzó C, Escudero-García D, Tosca J, Ríos M-P, Montón C, Durbán L, Ballester J, Benlloch S, Urios A, San-Miguel T, Kosenko E, Serra M-Á, Felipo V, Montoliu C (2022). Metabolic syndrome is associated with poor response to rifaximin in minimal hepatic encephalopathy. Sci Rep.

[CR9] Balzano T, Forteza J, Borreda I, Molina P, Giner J, Leone P, Urios A, Montoliu C, Felipo V (2018). Histological Features of Cerebellar Neuropathology in Patients With Alcoholic and Nonalcoholic Steatohepatitis. J Neuropathol Exp Neurol.

[CR10] Basu S, Zethelius B, Helmersson J, Berne C, Larsson A, Arnlöv J (2011). Cytokine-mediated inflammation is independently associated with insulin sensitivity measured by the euglycemic insulin clamp in a community-based cohort of elderly men. Int J Clin Exp Med.

[CR11] Bélanger M, Allaman I, Magistretti PJ (2011). Brain energy metabolism: focus on astrocyte-neuron metabolic cooperation. Cell Metab.

[CR12] Biessels GJ, Staekenborg S, Brunner E, Brayne C, Scheltens P (2006). Risk of dementia in diabetes mellitus: a systematic review. Lancet Neurol.

[CR13] Biessels GJ, Strachan MWJ, Visseren FLJ, Kappelle LJ, Whitmer RA (2014). Dementia and cognitive decline in type 2 diabetes and prediabetic stages: towards targeted interventions. The Lancet Diabetes & Endocrinology.

[CR14] Biessels GJ, Despa F (2018). Cognitive decline and dementia in diabetes mellitus: mechanisms and clinical implications. Nat Rev Endocrinol.

[CR15] Butt Z, Jadoon NA, Salaria ON, Mushtaq K, Riaz IB, Shahzad A, Hashmi AM, Sarwar S (2013). Diabetes mellitus and decompensated cirrhosis: risk of hepatic encephalopathy in different age groups. J Diabetes.

[CR16] Campagna F, Montagnese S, Ridola L, Senzolo M, Schiff S, de Rui M, Pasquale C, Nardelli S, Pentassuglio I, Merkel C, Angeli P, Riggio O, Amodio P (2017). The animal naming test: An easy tool for the assessment of hepatic encephalopathy. Hepatology.

[CR17] Chow LS, Albright RC, Bigelow ML, Toffolo G, Cobelli C, Nair KS (2006). Mechanism of insulin’s anabolic effect on muscle: measurements of muscle protein synthesis and breakdown using aminoacyl-tRNA and other surrogate measures. Am J Physiol Endocrinol Metab.

[CR18] Colognesi M, Gabbia D, de Martin S (2020) Depression and Cognitive Impairment-Extrahepatic Manifestations of NAFLD and NASH. 10.3390/biomedicines8070229. Biomedicines 8 10.3390/biomedicines8070229 PMC7400092 32708059

[CR19] Cordoba J, Ventura-Cots M, Simón-Talero M, Amorós À, Pavesi M, Vilstrup H, Angeli P, Domenicali M, Ginés P, Bernardi M, Arroyo V (2014). Characteristics, risk factors, and mortality of cirrhotic patients hospitalized for hepatic encephalopathy with and without acute-on-chronic liver failure (ACLF). J Hepatol.

[CR20] Diaz J, Monge E, Roman R, Ulloa V (2008). Diabetes as a risk factor for infections in cirrhosis. Am J Gastroenterol.

[CR21] Diaz-Gutierrez FL, Ladero JM, Diaz-Rubio M (1998). Acarbose-induced acute hepatitis. Am J Gastroenterol.

[CR22] Elkrief L, Chouinard P, Bendersky N, Hajage D, Larroque B, Babany G, Kutala B, Francoz C, Boyer N, Moreau R, Durand F, Marcellin P, Rautou P-E, Valla D (2014). Diabetes mellitus is an independent prognostic factor for major liver-related outcomes in patients with cirrhosis and chronic hepatitis C. Hepatology.

[CR23] Elkrief L, Rautou P-E, Sarin S, Valla D, Paradis V, Moreau R (2016). Diabetes mellitus in patients with cirrhosis: clinical implications and management. Liver Int.

[CR24] Fadel JR, Reagan LP (2016). Stop signs in hippocampal insulin signaling: the role of insulin resistance in structural, functional and behavioral deficits. Curr Opin Behav Sci.

[CR25] Feinkohl I, Price JF, Strachan MWJ, Frier BM (2015). The impact of diabetes on cognitive decline: potential vascular, metabolic, and psychosocial risk factors. Alzheimers Res Ther.

[CR26] Foerster F, Gairing SJ, Müller L, Galle PR (2022). NAFLD-driven HCC: Safety and efficacy of current and emerging treatment options. J Hepatol.

[CR27] Forton DM, Allsop JM, Main J, Foster GR, Thomas HC, Taylor-Robinson SD (2001). Evidence for a cerebral effect of the hepatitis C virus. Lancet.

[CR28] Friedman SL, Neuschwander-Tetri BA, Rinella M, Sanyal AJ (2018). Mechanisms of NAFLD development and therapeutic strategies. Nat Med.

[CR29] Gairing SJ, Anders J, Kaps L, Nagel M, Michel M, Kremer WM, Hilscher M, Galle PR, Schattenberg JM, Wörns M-A, Labenz C (2022). Evaluation of IL-6 for Stepwise Diagnosis of Minimal Hepatic Encephalopathy in Patients With Liver Cirrhosis. Hepatol Commun.

[CR30] Gairing SJ, Müller L, Kloeckner R, Galle PR, Labenz C (2022). Review article: post-TIPSS hepatic encephalopathy-current knowledge and future perspectives. Aliment Pharmacol Ther.

[CR31] Gentile S, Guarino G, Romano M, Alagia IA, Fierro M, Annunziata S, Magliano PL, Gravina AG, Torella R (2005). A randomized controlled trial of acarbose in hepatic encephalopathy. Clin Gastroenterol Hepatol.

[CR32] Goldbecker A, Weissenborn K, Hamidi Shahrezaei G, Afshar K, Rümke S, Barg-Hock H, Strassburg CP, Hecker H, Tryc AB (2013). Comparison of the most favoured methods for the diagnosis of hepatic encephalopathy in liver transplantation candidates. Gut.

[CR33] Grover VPB, Southern L, Dyson JK, Kim JU, Crossey MME, Wylezinska-Arridge M, Patel N, Fitzpatrick JA, Bak-Bol A, Waldman AD, Alexander GJ, Mells GF, Chapman RW, Jones DEJ, Taylor-Robinson SD (2016). Early primary biliary cholangitis is characterised by brain abnormalities on cerebral magnetic resonance imaging. Aliment Pharmacol Ther.

[CR34] Gundling F, Seidl H, Strassen I, Haller B, Siegmund T, Umgelter A, Pehl C, Schepp W, Schumm-Draeger PM (2013). Clinical manifestations and treatment options in patients with cirrhosis and diabetes mellitus. Digestion.

[CR35] Heni M, Schöpfer P, Peter A, Sartorius T, Fritsche A, Synofzik M, Häring H-U, Maetzler W, Hennige AM (2014). Evidence for altered transport of insulin across the blood-brain barrier in insulin-resistant humans. Acta Diabetol.

[CR36] Horvath TL, Sarman B, García-Cáceres C, Enriori PJ, Sotonyi P, Shanabrough M, Borok E, Argente J, Chowen JA, Perez-Tilve D, Pfluger PT, Brönneke HS, Levin BE, Diano S, Cowley MA, Tschöp MH (2010). Synaptic input organization of the melanocortin system predicts diet-induced hypothalamic reactive gliosis and obesity. Proc Natl Acad Sci U S A.

[CR37] Jepsen P, Ott P, Andersen PK, Sørensen HT, Vilstrup H (2010). Clinical course of alcoholic liver cirrhosis: a Danish population-based cohort study. Hepatology.

[CR38] Jepsen P, Watson H, Andersen PK, Vilstrup H (2015). Diabetes as a risk factor for hepatic encephalopathy in cirrhosis patients. J Hepatol.

[CR39] Kalaitzakis E, Olsson R, Henfridsson P, Hugosson I, Bengtsson M, Jalan R, Björnsson E (2007). Malnutrition and diabetes mellitus are related to hepatic encephalopathy in patients with liver cirrhosis. Liver Int.

[CR40] Kjærgaard K, Mikkelsen ACD, Wernberg CW, Grønkjær LL, Eriksen PL, Damholdt MF, Mookerjee RP, Vilstrup H, Lauridsen MM, Thomsen KL (2021) Cognitive Dysfunction in Non-Alcoholic Fatty Liver Disease-Current Knowledge, Mechanisms and Perspectives. J Clin Med 10. 10.3390/jcm10040673 10.3390/jcm10040673 PMC7916374 33572481

[CR41] Kullmann S, Kleinridders A, Small DM, Fritsche A, Häring H-U, Preissl H, Heni M (2020). Central nervous pathways of insulin action in the control of metabolism and food intake. The Lancet Diabetes & Endocrinology.

[CR42] Kulzer B, Albus C, Herpertz S, Kruse J, Lange K, Lederbogen F, Petrak F (2013). Psychosoziales und Diabetes (Teil 1). Diabetol und Stoffwechsel.

[CR43] La Vega J, Crespo M, Escudero JM, Sánchez L, Rivas LL (2000) Hepatitis aguda por acarbosa. Descripción de 2 episodios en una misma paciente (Acarbose-induced acute hepatitis. Report of two events in the same patient). Gastroenterol Hepatol 23:282–284. https://doi.org/Case 15324623

[CR44] Labenz C, Baron JS, Toenges G, Schattenberg JM, Nagel M, Sprinzl MF, Nguyen-Tat M, Zimmermann T, Huber Y, Marquardt JU, Galle PR, Wörns M-A (2018). Prospective evaluation of the impact of covert hepatic encephalopathy on quality of life and sleep in cirrhotic patients. Aliment Pharmacol Ther.

[CR45] Labenz C, Toenges G, Huber Y, Nagel M, Marquardt JU, Schattenberg JM, Galle PR, Labenz J, Wörns M-A (2019). Raised serum Interleukin-6 identifies patients with liver cirrhosis at high risk for overt hepatic encephalopathy. Aliment Pharmacol Ther.

[CR46] Labenz C, Nagel M, Kremer WM, Hilscher M, Schilling CA, Toenges G, Kuchen R, Schattenberg JM, Galle PR, Wörns M-A (2020). Association between diabetes mellitus and hepatic encephalopathy in patients with cirrhosis. Aliment Pharmacol Ther.

[CR47] Labenz C, Toenges G, Schattenberg JM, Nagel M, Huber Y, Marquardt JU, Labenz J, Galle PR, Wörns M-A (2020). Outcome Prediction of Covert Hepatic Encephalopathy in Liver Cirrhosis: Comparison of Four Testing Strategies. Clin Transl Gastroenterol.

[CR48] Lauridsen MM, Poulsen L, Rasmussen CK, Høgild M, Nielsen MK, de Muckadell OBS, Vilstrup H (2016). Effects of common chronic medical conditions on psychometric tests used to diagnose minimal hepatic encephalopathy. Metab Brain Dis.

[CR49] Mogi M, Horiuchi M (2011). Neurovascular coupling in cognitive impairment associated with diabetes mellitus. Circ J.

[CR50] Morris JK, Vidoni ED, Honea RA, Burns JM (2014). Impaired glycemia increases disease progression in mild cognitive impairment. Neurobiol Aging.

[CR51] Mosher VAL, Swain MG, Pang JXQ, Kaplan GG, Sharkey KA, MacQueen GM, Goodyear BG (2018). Magnetic resonance imaging evidence of hippocampal structural changes in patients with primary biliary cholangitis. Clin Transl Gastroenterol.

[CR52] Nagel M, Weidner V, Schulz S, Marquardt JU, Galle PR, Schattenberg JM, Nguyen-Tat M, Wörns M-A, Labenz C (2022). Continued alcohol consumption and hepatic encephalopathy determine quality of life and psychosocial burden of caregivers in patients with liver cirrhosis. Health Qual Life Outcomes.

[CR53] Nardelli S, Lattanzi B, Merli M, Farcomeni A, Gioia S, Ridola L, Riggio O (2019). Muscle Alterations Are Associated With Minimal and Overt Hepatic Encephalopathy in Patients With Liver Cirrhosis. Hepatology.

[CR54] Praktiknjo M, Simón-Talero M, Römer J, Roccarina D, Martínez J, Lampichler K, Baiges A, Low G, Llop E, Maurer MH, Zipprich A, Triolo M, Maleux G, Fialla AD, Dam C, Vidal-González J, Majumdar A, Picón C, Toth D, Darnell A, Abraldes JG, López M, Jansen C, Chang J, Schierwagen R, Uschner F, Kukuk G, Meyer C, Thomas D, Wolter K, Strassburg CP, Laleman W, La Mura V, Ripoll C, Berzigotti A, Calleja JL, Tandon P, Hernandez-Gea V, Reiberger T, Albillos A, Tsochatzis EA, Krag A, Genescà J, Trebicka J (2020). Total area of spontaneous portosystemic shunts independently predicts hepatic encephalopathy and mortality in liver cirrhosis. J Hepatol.

[CR55] Qin J, Li Y, Cai Z, Li S, Zhu J, Zhang F, Liang S, Zhang W, Guan Y, Shen D, Peng Y, Zhang D, Jie Z, Wu W, Qin Y, Xue W, Li J, Han L, Lu D, Wu P, Dai Y, Sun X, Li Z, Tang A, Zhong S, Li X, Chen W, Xu R, Wang M, Feng Q, Gong M, Yu J, Zhang Y, Zhang M, Hansen T, Sanchez G, Raes J, Falony G, Okuda S, Almeida M, LeChatelier E, Renault P, Pons N, Batto J-M, Zhang Z, Chen H, Yang R, Zheng W, Li S, Yang H, Wang J, Ehrlich SD, Nielsen R, Pedersen O, Kristiansen K, Wang J (2012). A metagenome-wide association study of gut microbiota in type 2 diabetes. Nature.

[CR56] Reuter B, Walter K, Bissonnette J, Leise MD, Lai J, Tandon P, Kamath PS, Biggins SW, Rose CF, Wade JB, Bajaj JS (2018). Assessment of the spectrum of hepatic encephalopathy: A multicenter study. Liver Transpl.

[CR57] Rose CF, Amodio P, Bajaj JS, Dhiman RK, Montagnese S, Taylor-Robinson SD, Vilstrup H, Jalan R (2020). Hepatic encephalopathy: Novel insights into classification, pathophysiology and therapy. J Hepatol.

[CR58] Sharma G, Parihar A, Talaiya T, Dubey K, Porwal B, Parihar MS (2020) Cognitive impairments in type 2 diabetes, risk factors and preventive strategies. J Basic Clin Physiol Pharmacol 31. 10.1515/jbcpp-2019-0105 10.1515/jbcpp-2019-0105 31967962

[CR59] Sigal SH, Stanca CM, Kontorinis N, Bodian C, Ryan E (2006). Diabetes mellitus is associated with hepatic encephalopathy in patients with HCV cirrhosis. Am J Gastroenterol.

[CR60] Spengler U, Stellaard F, Ruckdeschel G, Scheurlen C, Kruis W (1989). Small intestinal transit, bacterial growth, and bowel habits in diabetes mellitus. Pancreas.

[CR61] Stewart R, Liolitsa D (1999). Type 2 diabetes mellitus, cognitive impairment and dementia. Diabet Med.

[CR62] Tinajero MG, Malik VS (2021). An Update on the Epidemiology of Type 2 Diabetes: A Global Perspective. Endocrinol Metab Clin North Am.

[CR63] Tortelli R, Lozupone M, Guerra V, Barulli MR, Imbimbo BP, Capozzo R, Grasso A, Tursi M, Di Dio C, Sardone R, Giannelli G, Seripa D, Misciagna G, Panza F, Logroscino G (2017). Midlife Metabolic Profile and the Risk of Late-Life Cognitive Decline. J Alzheimers Dis.

[CR64] Trail KC, Stratta RJ, Larsen JL, Ruby EI, Patil KD, Langnas AN, Donovan JP, Sorrell MF, Zetterman RK, Pillen TJ (1993). Results of liver transplantation in diabetic recipients. Surgery.

[CR65] Tranah TH, Vijay GKM, Ryan JM, Shawcross DL (2013). Systemic inflammation and ammonia in hepatic encephalopathy. Metab Brain Dis.

[CR66] van der Heide LP, Kamal A, Artola A, Gispen WH, Ramakers GMJ (2005). Insulin modulates hippocampal activity-dependent synaptic plasticity in a N-methyl-d-aspartate receptor and phosphatidyl-inositol-3-kinase-dependent manner. J Neurochem.

[CR67] Vilstrup H, Amodio P, Bajaj J, Cordoba J, Ferenci P, Mullen KD, Weissenborn K, Wong P (2014). Hepatic encephalopathy in chronic liver disease: 2014 Practice Guideline by the American Association for the Study of Liver Diseases and the European Association for the Study of the Liver. Hepatology.

[CR68] Wegener M, Börsch G, Schaffstein J, Luerweg C, Leverkus F (1990). Gastrointestinal transit disorders in patients with insulin-treated diabetes mellitus. Dig Dis.

[CR69] Weinstein AA, de Avila L, Paik J, Golabi P, Escheik C, Gerber L, Younossi ZM (2018). Cognitive Performance in Individuals With Non-Alcoholic Fatty Liver Disease and/or Type 2 Diabetes Mellitus. Psychosomatics.

[CR70] Weissenborn K, Heidenreich S, Giewekemeyer K, Rückert N, Hecker H (2003). Memory function in early hepatic encephalopathy. J Hepatol.

[CR71] Weissenborn K (2019). Minimal/Covert Hepatic Encephalopathy - Impact of Comorbid Conditions. J Clin Exp Hepatol.

[CR72] Whitfield JB, Schwantes-An T-H, Darlay R, Aithal GP, Atkinson SR, Bataller R, Botwin G, Chalasani NP, Cordell HJ, Daly AK, Day CP, Eyer F, Foroud T, Gleeson D, Goldman D, Haber PS, Jacquet J-M, Liang T, Liangpunsakul S, Masson S, Mathurin P, Moirand R, McQuillin A, Moreno C, Morgan MY, Mueller S, Müllhaupt B, Nagy LE, Nahon P, Nalpas B, Naveau S, Perney P, Pirmohamed M, Seitz HK, Soyka M, Stickel F, Thompson A, Thursz MR, Trépo E, Morgan TR, Seth D (2022). A genetic risk score and diabetes predict development of alcohol-related cirrhosis in drinkers. J Hepatol.

[CR73] Wlazlo N, Beijers HJBH, Schoon EJ, Sauerwein HP, Stehouwer CDA, Bravenboer B (2010). High prevalence of diabetes mellitus in patients with liver cirrhosis. Diabet Med.

[CR74] You Y, Liu Z, Chen Y, Xu Y, Qin J, Guo S, Huang J, Tao J (2021). The prevalence of mild cognitive impairment in type 2 diabetes mellitus patients: a systematic review and meta-analysis. Acta Diabetol.

[CR75] Zilliox LA, Chadrasekaran K, Kwan JY, Russell JW (2016). Diabetes and Cognitive Impairment. Curr Diab Rep.

